# From re-pair and re-production to (*re)generation*: bio-objects as indicators of cultural change

**DOI:** 10.3325/cmj.2012.53.502

**Published:** 2012-10

**Authors:** Bettina Bock von Wülfingen

**Affiliations:** Institute for Cultural History and Theory, Humboldt University of Berlin, Berlin, Germany Member of working group 3 “generative relations”

Since over a decade and ever more often we observe a clash between specific “new” living entities and the governance and regulation of (bio-)medical practices. These are the entities we call “bio-objects.” As will become clearer in the following, these bio-objects are indicators of a fundamental change between different systems of thought and practice: this is in the case of medicine the change from reproduction and repair to regeneration. This change in medicine is part of a broader cultural change.

## Who is the actor in bio-objectification?

In an earlier article, this COST network described bio-objects as creatures that have been made at the work benches of the life sciences, such as genetically-modified organisms or transpecies animals, as well as entities that we are already familiar with but which have been brought into new spaces, such as stem cells, which were removed from the cord blood after delivery and stored in cord blood banks, or in vitro fertilization (IVF) embryos that dwell in Petri dishes in laboratories (1).

What we should stress additionally is that an entity can only turn into a bio-object if being an interrelated entity. Only in the interaction with its environment (eg, IVF-patients, EU regulators, media discourse) can it turn out to be problematic – meaning that it is a challenge to common ways of dealing with living entities. The bio-object is not problematic in itself but in *relation to* our existing knowledge and practices (2). This means that the entity is understood as bio-object *together* with the challenged regulative body, and *together* with some observing entity, who certifies that there lies a conflict in their interrelation and builds a bio-objectifying apparatus through their interaction (3).

## “Normal” objects (non bio-objects) are related to specific historic environment

**Figure 1:**
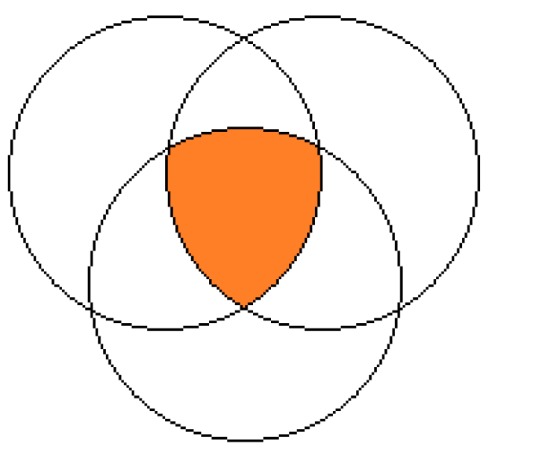
Bio-objectifying apparatus. The gray field symbolizes the bio-object.

As shown in the Figure 1, not all objects generated in laboratories or scientific discourse turn into bio-objects. They only do so when they need to receive a new identity (4) in order to fit current regulations (as the term “pre-embryo” in some countries shows) or when there’s enough interest in changing regulations or in changing the moral economy regarding the bio-object (as with preimplantation diagnosis, or the different, newly emerging types of mothering by egg cell donation).

An excursion into the history of genetics might make this interdependency of history and object more plausible. It was in the 1880s to 1890s that it became common sense between a group of researchers that only the nucleus carried the substances of inheritance and not the complete cell. The identification of the cell-nuclear substances (later called desoxy-ribo-nuclein-acids, DNA) as *the* substance of inheritance is an idea deeply entangled with the history of the foundation of the German nation (5). Naturalists working in the German lands such as Oscar Hertwig, Carl Wilhelm von Nägeli, August Weismann, and others conceptualized conception and heredity as processes governed by rules identical to first drafts of the then new German Civil Law Code. They divided a reproductive sphere (the “nutritive” plasma, similar to the private, non-economic part of the civil household) from a productive sphere (the nucleus), where economic riches were amassed for the future and managed correspondingly. Quite as the Civil Law Code ordered for the civil household, the nutritive part of the cellular household was perceived as female (stemming from the egg cell) and the productive part was perceived as male. According to the law, the family father had the say over all family members’ economy. This concept of a division – between mere nutritive plasma on one hand and managing and inheritance functions of the nucleus on the other – as adapted to the cell was ridiculed and fought against by researchers in the United States. Still it soon spread and during the 20th century became the dominant model framework for genetics. Thus, the idea of a transmission of capital according to specific rules (of inheritance) and of its management in the cell surfaced at a seldom historic moment: the moment when not only natural scientists but simultaneously the rest of the German civil society renegotiated rules for living together and for the transmission of property between family members. This shows that the concept of this specific location of genetic substance in the nucleus, later taken for granted, resulted from the stabilization of a highly *improbable* constellation at one place at a specific historical moment: it was an improbable constellation as two seldom developments became entangled and reinforced each other.

## Bio-objectification is a destabilization of “highly improbable” historical constellations

The historical concept of reproduction meant sexual generation (ie, the meeting of two unrelated entities conceptualized as very different from each other, resulting in a third entity). In the past circa 130 years, this was understood as the most important way to generate life, in contrast to what we see today with the rising acceptance of the idea that (sexual) reproduction is only one way of many, and that the overwhelming majority of cells including most organisms do some kind of off-budding (6-8). Simultaneously new scientific models appear that integrate plasma and nucleus in inheritance. Medicine, meanwhile, also experiences a turn from repair (a machinist concept) to regeneration.

The above example at the core of biology teaches us that we can understand bio-objectification as a process in which these “highly improbable” objects are destabilized again. This can happen when, for instance, through globalization they meet with concepts that don’t pertain to the European enlightenment and modernity. The destabilization can also take place when such a model reaches its explanatory limits: this happened when the Human Genome Project did not render the expected knowledge and had to leave it to epigenetics and (epi-) genomics to fit DNA into its cellular context.

## Bio-objects indicate cultural change

As is often mentioned and again repeated in this article, bio-objects are not easy to tackle. In contrast, it is a typical feature of bio-objects to cause uneasiness in humans and to mess with definitions and laws. But why then and under what circumstances do we allow bio-objects to behave this way?

Most entities that we call bio-objects are the result of some medico-scientific procedure which often puts matter “out of place.” So, are bio-objects just the result of a technical feasibility that we did not have earlier?

Examples of non-universality of bio-objects show that this is not the case. The same entity can be a bio-object in one country but not in another. For example in countries where cloning or any handling of human embryos is prohibited (as in Germany until recently) and where (economic or moral) interest or need for cloning does not enter any audible discourse, the bio-object “cloned embryo” would not come into existence.

Instead of just being the result of technological changes, the willingness to perceive and allow the clash of specific entities with our conventional modern regulations has risen.

We can make out at least two reasons for this change:

1. Discontent with modern binaries and interest in complexity. All of these bio-objects that live today in the borderland between nature and culture, between human and animal, between self and non-self had progenitors: they are not new. There were chimeras all around, we are all composed of cells from different individuals (our mother’s and bacteria, just to name a few); clones existed before as well as hermaphrodites and if we had wanted, we could have known that genes are not static a long time ago. But it is recently in the past decades that the discontent with modern binaries, which often favored one type of modern subject and not the other, had risen and made the inbetweens more visible and laudable. The rise of the bio-object indicates a crisis of those dichotomies that were meant to help organize the world in the past centuries.

2. Profitability of the border-crossing. Even if the bio-object itself is not intrinsically new, the ability to generate it and the willingness to request services and specific products out of laboratory entities that challenge our common understanding of nature and culture is rather recent. Next to the biotic entity itself and to the regulation/discourse that it meets (Figure 1), it is this (economic) interest that helps the bio-object to emerge. Therefore, the bio-object is always entangled with issues of justice – when we think of insurance practices, distribution of medical resources, or ownership of organic or bio-virtual material.

To understand the hybridity of techniques involved even in these economic processes, it is helpful to remember that the profitability of bio-objects recently took off through the combination of genomics with reproductive sciences and techniques: the decoding of DNA/RNA has often been called “reading” the genome. Correspondingly, when technical engineering found out how to change DNA, this was called “writing.” A whole “economy of hope” was built around these techniques. But it was not the ability to re-write or “write” the DNA that opened up the new options for a regenerative medicine on the horizon today, including stem cells therapy or cybrids. Instead the connection between genetic/genomic knowledge with its complex environment in the cell, as well as with techniques stemming from the reproductive (farm) practices/sciences, rendered it a useful tool of research and, maybe once, for therapy. The social anthropologist Sarah Franklin used the notion of the “embryo flap” to visualize this border crossing from therapy to research and their respective economies: the embryo flap links the IVF-surgery with the compound for stem cell research behind (9).

These conditions confront us with new problems. The interest in the inbetweens and the recent distrust in easier to understand binaries seems to go hand in hand with the higher levels of complexity reached in biomedical modeling these days (see reason for cultural change number 1). On the other hand (see reason for cultural change number 2), research in social studies and philosophy of science seems to indicate that in order for knowledge or product to reach “marketability” (such as publishing or patenting) its complexity needs to be reduced (10,11).

This means that if we don’t want to allow the market-driven reduction of complexity and if we want, instead, to follow a rising interest in complexity (combination of number 1 and 2 above), we need measures to tackle the problem of the marketability of complexity.
